# Semiconductor Nanocrystal Quantum Dot Synthesis Approaches Towards Large-Scale Industrial Production for Energy Applications

**DOI:** 10.1186/s11671-015-1166-y

**Published:** 2015-12-04

**Authors:** Michael Z. Hu, Ting Zhu

**Affiliations:** Oak Ridge National Laboratory, Oak Ridge, TN37831-6181 USA

**Keywords:** Quantum dots, QDs, Scale up, Synthesis, Production

## Abstract

This paper reviews the experimental synthesis and engineering developments that focused on various green approaches and large-scale process production routes for quantum dots. Fundamental process engineering principles were illustrated. In relation to the small-scale hot injection method, our discussions focus on the non-injection route that could be scaled up with engineering stir-tank reactors. In addition, applications that demand to utilize quantum dots as “commodity” chemicals are discussed, including solar cells and solid-state lightings.

## Review

Semiconductor nanocrystal quantum dots have attracted more and more interests in solar cell, solid-state lighting, and biological labeling fields due to the unique size-tunable light absorption and emission properties. The large quantity demands of high-quality quantum dots for advanced energy applications require an industrial applicable production method. However, the current quantum dot (QD) synthesis methods can only fulfill the requirements of small-scale Research and Development (R&D) and biological sampling/imaging. Novel approaches of QD synthesis suitable for scale-up production are thus essential for the commercialization of optoelectronic devices in the near future. This review paper discusses various synthesis methods for semiconductor nanocrystal quantum dots and their potential for future industrial scale-up. To do this, an insight view of the available synthesis mechanisms is also presented to help in identifying the controlling factor in scale-up.

Here, the quantum dots are defined as the semiconductor nanocrystals with the quantum confinement. Thus, the semiconductor nanoparticles with dimensions exceeding the Bohr radius are not within our discussion since their application deviates from the quantum tunability from the quantum confinement effect. Also, the discussion of synthesis only restricts to those quantum dots which have identified their applications in energy saving and utilization fields; materials with special morphology but without confirmed properties suitable for those applications are not within the scope of this review. Metal oxide semiconductor such as ZnO, due to their distinct properties and applications and the large amount of literatures available, will not be discussed as well (readers may refer to Ref. [1] for additional information). Due to the aim of practical industry application, the authors also would like to restrict the discussion within the material system with high quality suitable for energy applications, namely monodisperse with stable surface protection, decent optical or optoelectronic properties.

There are a number of literatures available for the synthesis of semiconductor nanocrystals or large-scale synthesis of nanoparticles. For example, Ref. [1] has provided a comprehensive introduction of nanoparticle production in large volume covering elemental metals and metalloids (semiconductors), chalcogenide II–VI and IV–VI semiconductors, III–V semiconductors, and oxides. The review will try to include the most recent updates not included in those reviews and discuss the most feasible approaches towards large-scale production in a practical point of view.

Since the large-scale synthesis is aimed towards advanced energy application, the potential candidate must feature or have the potential to fulfill the following requirements:

1. Easy processing
2. High reproducibility
3. Low cost
4. Environmental friendly

The most widely used QD synthesis method for high-quality QD production is the hot injection approach. There are a few number of review articles in the literature for the discussion of the injection method [47]. This approach features a fast injection of precursor into a hot solution containing another precursor and has been successfully achieved in various systems. However, the reaction requires an instant homogeneous reaction which is hard to achieve in large volume reaction vessels. This also brings an inherent complication and difficulties in reproduction. Thus, the injection approach is not suitable for scale-up and large quantity synthesis.

### Non-Injection Organic Synthesis

The most challenging part of QD synthesis is the way to initiate reaction. A monodispersed quantum dot needs the formation of a uniform nanocrystal nucleus in a very short period of time. This can be achieved by fast injection of one precursor into the solution to start the fast and homogeneous nucleus formation. This has by far been proved as the most successful approach in various QD families. But the homogeneous reaction initiated by fast injection is difficult to achieve in large volume reaction vessels. The special requirement of fast and homogeneous reaction is not suitable for industrial large-scale chemical vessel.

Due to the inherent limitation of injection approach, non-injection nanocrystal synthesis method has been developed by various groups. Contrary to the injection approach, two different precursors are present in the system simultaneously before the reaction starts at a certain temperature. As indicated in the injection approach, clear separation between nucleation and growth is desired for the production of monodispersed QD. The colloidal nanocrystals usually grow at an elevated temperature which requires a heating process with a certain temperature growth rate. The heating process could initiate active precursors to nucleate partially and result in a concurrent nucleation and growth [19]. On the other hand, for the precursors with too low activities, very little amount of nuclei will form and the growth rate can be too fast to control. As suggested, one ideal solution is the selection of an appropriate chemical to initiate nucleation at a desired temperature. This concept was proved in CdS system by Cao and his collaborators [2]. To do this, two nucleation initiators were introduced in a CdS synthesis system, tetraethylthiuram disulfides and 2,2′-dithiobisbenzothiazole, namely I1 and I2. The precursors and solvents used are the same as those used in the injection approach, with cadmium myristate and sulfur as the two precursors, with myristic acid and octadecene (ODE) serving as the capping ligand and solvent, respectively. The system was preheated to 120 °C under vacuum to obtain a clear solution. After that, the temperature was slowly increased to 240 °C for nuclei to start. The size distribution narrows down until 4 min of growth and can be maintained for growth time of longer than 12 h without new nucleation detected. The absorption and photoluminescence (PL) of the CdS nanocrystals are comparable to the product obtained by injection approaches [19, 90, 91]. Although a noticeable surface-trap emission is accompanied in the PL, it can be removed by gentle fluorescent illuminations. The crystal structure is found to be zinc blende instead of wurtzite from injection approaches.

The nucleation initiators are not available for all material systems. A more practical way is the selection of appropriate precursors with appropriate activities. By carefully controlling the available precursor concentration and activity in the solution, the monodispersed QD can also be obtained by a non-injection method. Ideal precursors should exhibit significant reactivity transition near the desired growth temperature, in other words, almost no reactivity below the point and very high reactivity above the point. Some of the features helping the transition include the melting/decomposition point and solubility change under different temperatures. A few appropriate precursors and solvents selected are the following: cadmium myristate and selenium powder in ODE for CdSe spherical nanocrystals, cadmium myristate and tributylphophine selenice (TBPSe) in ODE for CdSe nonspherical nanocrystals, cadmium octadecylphosphonate and TBPTe for CdTe quantum dots [3]. Both CdSe and CdTe nanocrystals synthesized by this approach show sizes with a standard deviation of less than 5 % [3]. The typical CdSe nanocrystals synthesized have a PL quantum yield of 30–40 % and show a zinc blende structure [3].

Similar to the non-injection approaches above, Yu and her collaborators developed various families of II–VI binary and ternary magic-sized nanocrystals (MSNs) [4–10]. The magic-sized nanocrystals are considered thermodynamically favorable due to their special space structure such as core-shell configuration. More importantly, magic-sized nanocrystals are single-sized ensembles and featuring extremely narrow absorption/emission width. These special optical properties are very attractive for solid-state lighting and telecommunication applications.

The II–VI binary systems Yu has developed include CdSe [4, 5], CdS [6], and PbS [7] magic-sized or regular-sized quantum dots (MSQDs). For CdSe MSQD, the cadmium source is cadmium acetate dehydrate (Cd(OAc)_2_ · 2H_2_O) and elemental Se as the selenium source compound. The reaction temperature ranges from 120 to 240 °C with three families of MSQD produced [5]. Depending on the wavelength of bandgap absorption, they are termed as Family 395,463, and 513 nm, respectively. The synthesized QDs exhibit bright photoluminescence with a full width at half maximum (FWHM) of 8 to 10 nm. The Stokes shift is also smaller than the regular QD, e.g., Family 463 has an emission peak at 465 nm rending a Stokes shift of only 2 nm. This small Stokes shift might indicate a dominant band-edge emission with very little trap emission involved. 1-Octadecene is used as the reaction medium and fatty acids are used as the capping ligands. The success of the non-injection approach lies in the low activity of the cadmium precursor. The precursor is in the form of Cd(OAc)_x_–(OOC–(CH_2_)_n_–CH_3_)_2 − x_ and releases cadmium slowly for the reaction. A low acid-to-Cd feed molar ratio is important to keep the low cadmium precursor activity. For different kinds of fatty acids, an optimum window of ligand length is also present (C12–C18). A longer ligand is a better soluble in ODE and weaker in binding with cadmium. Correspondingly, longer ligands will increase the activity of Cd precursor and monomer, thus leading to large MSQDs. Besides the nature of acids and acid-to-Cd ratio, the reaction temperature and Cd-to-Se feed molar ratio are also investigated as affecting factors. High Cd-to-Se ratio is found to prevent the dissociation of the formed MSQDs. In one word, a reaction temperature of 200–240 °C, a low acid-to-Cd ratio, a Cd-to-Se ratio, and a ligand length of C12 to C18 are considered optimum for producing CdSe MSQD with high nanocrystal concentration and PL QY.

A similar reaction can be carried out in CdS system with elemental S as the sulfur source [6]. Myristic acid (MA) is used as a capping ligand, and 2,2′-dithiobisbenzothiazole (MBTS) is added to promote sulfur reactivity. A series of experiment conditions are also investigated to find out the optimized synthesis condition. With the acid-to-Cd ratio fixed (2:1), a ratio of (1–2)Cd/1S and (8–32)S/1MBTS, a growth temperature of 220–350 °C is determined to be ideal for QD growth. The obtained CdS QD has a FWHM of 17–22 nm and a quantum yield up to 30 %.

A similar non-injection and low temperature approach was also used for PbS QD synthesis [7]. The obtained PbS QD has a bandgap emission from 600 to 900 nm with a narrow emission FWHM of 100 nm. Different lead and sulfur sources have been used including lead oxide, lead acetate, bis(trimethylsilyl)sulfide ((TMS)2S), thioacetamide (TAA), and elemental sulfur (S). ODE is used as a reaction medium, and the reaction temperature is 30–120 °C.

Besides the binary system mentioned above, non-injection approaches have been proved successfully also in a number of ternary systems such as CdTeSe magic-sized nanocrystals and CdSeS regular nanocrystals. Both ternary systems use similar sources as the binary systems mentioned above, fatty acids as ligands and ODE as reaction medium. MBTS was added in CdSeS case for the purpose of activating sulfur. Solid-state 13C cross polarization/magic angle spinning (CP/MAS) NMR spectra combined with X-ray diffraction (XRD) confirmed the ternary alloyed system with uniformly distributed composition throughout the whole nanocrystals.

Besides the methods starting from two precursors as mentioned above, another non-injection approach uses a single-source precursor to start the direct synthesis of quantum dots. The earlier single-molecule precursor routes involve a hot injection of the precursor into TOPO solution at high temperature thus not favorable for large-scale synthesis [11, 12]. A recent work used lower temperature for mixing and higher temperature for reaction to avoid the hot injection step [13]. (Me_4_N)_4_[S_4_Cd_10_(SPh)_16_] is the single-source precursor, and it was added into hexadecylamine (HDA) at 80 °C under argon before temperature increased to 230 °C for reaction. Large quantities (>10 g/L) of CdS QD could be produced by the method. A different approach using inorganic clusters as single-source precursor allows the nucleation starting at relatively low temperature. The technique is based on the introduction of an inorganic metal-chalcogenide cluster into an alkylamine solvent. In a typical reaction preparing CdSe, the precursor (Li)_4_[Cd_10_Se_4_(SPh)_16_] was added into HDA at 120 °C under argon before the whole system was raised to 220–240 °C for the reaction [14]. The single-source precursor is stable under ambient conditions, and the reactions can be readily scaled to large quantities (1–50 g/L) without substantial adjustment to the growth methodology. Via this approach, high-quality monodispersed CdSe, ZnSe, and CdSe/ZnS quantum dots have been prepared. One of the concerns for this method is the design and the synthesis of the single-source precursor. The additional requirement of preparing a single-source precursor and the limited availability of a suitable precursor for different material systems might restrict its further development.

### Synthesis Mechanisms

To achieve the scale-up synthesis of nanocrystals from current bench-based synthesis, it is important to understand the nanocrystal formation mechanism and the controlling factors. This could effectively form the guideline for the large-scale engineering production of nanocrystals.

For the regular QD synthesis, detailed mechanism investigations have been carried out [15, 19]. Usually, the QD formation contains the nucleation and growth process. The burst of nucleation is followed by a supersaturation of monomers. The growth of nuclei is then followed by consuming the additional monomers present in the system. Depending on the different monomer concentration, QD with different shapes can be grown with different size distributions. Both thermodynamics and kinetics contribute to the nanocrystal growth. For example, nanocrystals with low aspect ratios are obtained in the slow growth limit under thermodynamic control while the nanocrystals with highly anisotropic shapes require a kinetic growth regime. As Alivisatos and his colleagues proposed, at a relatively low monomer concentration, nearly round nanocrystals are formed with a relatively large size distribution. Under that regime, due to the depletion of smaller particles and the continuous growth of larger particles, a size “defocusing” or “Ostwald ripening” is observed. When the monomer concentration increases, nanocrystals with low aspect ratio are still observed with a narrower size distribution. This size “focusing” process is due to the faster growth rate of smaller particles than larger particles which finally results in a monodispersed nanocrystal ensemble. In the two regimes mentioned, the equilibrium shape of nanocrystals has a low aspect ratio since this minimized the surface area as well as the small energy difference between different facets. Since the growth rate of a facet depends exponentially on the surface energy, the high-energy facets will grow faster than low-energy facets. So when the monomer concentration continues to increase, kinetics will begin to control nanocrystal growth resulting nanocrystals with highly anisotropic shapes.

On the contrary, only nucleation seems to be involved in magic-sized nanocrystal formation. No growth in size can be observed after homogeneous nucleation of nanocrystal since the absorption peak position is fixed ever since it appears. We believe that the magic-sized QD formation is thermodynamically driven rather than kinetically controlled. Depending on the available experimental data, Yu and Hu have proposed that the formation of magic-sized QD is mainly determined by the thermodynamic equilibrium with little effect of chemical reaction kinetics involved [16]. By carefully controlling the equilibrium between precursors and nanocrystal products, different families of magic-sized QD and regular QD can be obtained. For example, a reaction window of 2.7MA-4Cd-1Se molar ratio, a Se concentration of 10 mmol/kg, and a reaction temperature of 200–240 °C could produce a magic-sized nanocrystal ensemble in pure form Family 463 (named by its bandgap absorption peak).

There are still arguments about the intrinsic properties of magic-sized quantum dots. Different morphologies have been observed under high-resolution TEM including dispersed particles and sheet structures. Magic-sized “cluster,” “platelets” [17] or “nanosheet” [18], and “quantum dot” have been used by different people, and the discrepancy needs to be justified by further experiment.

Some theoretical calculations have been carried out to prove that nanoclusters with magic numbers do exist with lower potential thus is thermodynamically more stable. So, it is plausible to assume that different magic-sized clusters have local minima in chemical potentials.

Previous investigations have noticed that magic-sized nanoclusters are frequently observed in the nucleation stage during the synthesis of elongated nanocrystals. The formation of magic-sized nanoclusters originates from their local minimum chemical potential because of the closed-shell configurations. As Peng and his collaborators suggested, magic-sized nanoclusters have local minimum chemical potential and form a local energy “well” in the figure of chemical potential vs the size [19]. The formation of magic-sized nanoclusters happens under a relatively high chemical potential and they are only stable at relatively high monomer concentrations due to their extremely small sizes. It is suggested that the magic-sized nanoclusters can undergo two pathways after the formation. With relatively high monomer concentration present in the reaction solution, it can grow into regular nanocrystals with larger size by “tunneling” through the lower thermodynamic barrier. On the other hand, it may decompose to monomers by “tunneling” thorough the high barrier on the reverse direction. This process is highly favored with a lower monomer concentration present in the solution.

It has also been observed that magic-sized clusters are formed only at the high monomer chemical potentials needed to form the rod-shaped nanocrystals, thus their chemical potential corresponds to local minima in the progression from precursors to final nanorods.

Jiang and Kelly tried to propose a mechanism to explain the formation of magic-sized clusters (MSCs) and to fit the current available experimental data [20]. A number of species are discussed in the system including the nuclei, the magic-sized clusters, nanorods (representing nanocrystals with highly anisotropic shapes), and nanospheres (nanocrystals with low aspect ratio). Instead of considering magic-sized cluster as a candidate of “critical size nuclei,” they differentiate the magic-sized clusters from the regular nuclei and proposed a chemical potential relation as μ_MSC_ (magic-sized cluster) > μ_N_ (nuclei) > μ_NR_ (nanorod) > μ_NS_ (nanosphere).

The central feature of the mechanism is the fast equilibrium between monomers and the magic-sized clusters. The magic-sized clusters can only form from monomers and solve back without the third possibility to form regular nuclei/particles. The monomer concentration has a saturation value of *M*_0_. The magic-sized clusters can only form when the monomer concentration exceeds *M*_0_ and will dissolve back when it is lower than the saturation value. The condensation and dissolution processes happen at a very fast rate so that magic-sized clusters serve as a reservoir for monomers. In this proposed mechanism, the possibility of magic-sized clusters serving as intermediate between monomer and regular nanorods is excluded.

Based on the chemical potential relation indicated above, several monomer concentration regimes can be identified. When the monomer concentration exceeds the saturation value *M*_0_, the additional monomer will form magic-sized clusters to keep monomer concentration within the saturation value *M*_0_. In this regime, μ_M_ (monomer chemical potential) = μ_MSC_ > μ_N_, only magic-sized clusters can be formed and they are in equilibrium with monomers. When the monomer concentration decreases below *M*_0_, no magic-sized clusters can be formed and the existing clusters will dissolve back to form monomers. At the same time, regular nanocrystal nuclei begin to form (μ_MSC_ > μ_M_ > μ_N_). When the monomer concentration continues to decrease (μ_N_ > μ_M_), no nucleation can occur and the additional monomer will attach to the available nuclei to continue the particle growth. If the monomer concentration continues to decrease, the reaction mechanism is identical to regular nanocrystal formation as explained above, with rod to sphere and Ostwald ripening (size “defocusing”) expected.

The above mechanism for magic-sized nanocluster formation is identical when the monomer concentration falls into the regular nanocrystal nucleation/formation regime. The formation of magic-sized clusters is due to the fairly high monomer concentration exceeding the “kinetic control” regime. The biggest difference between the two proposed mechanisms lies in the possibilities of magic-sized nanoclusters directly towards regular nanocrystals with larger size. Further well-designed experiments are expected to help elucidating the puzzle.

Theoretical calculations have been carried out to help understanding the MSQD forming mechanism. First-principle calculations using ultrasoft pseudopotentials and the generalized gradient approximation show that (CdSe)n to be cage-like polyhedral [21]. The Cd and Se ions will connect alternatively to form zigzag networks composed of four- and six-membered rings. Furthermore, the cage can be stabilized by filling with a core inside with connections to the cage. Thus, a selection of a highly symmetric cage with the right size of core will impose stringent restrictions for forming a stable nanostructure. By calculation, this novel 3D core-cage structure favors to take specific atom numbers to maximize the binding energy. For example, the smallest polar cages with the highest possible symmetry (octahedral) have the number of *n* = 12, 16, and 28, in which (CdSe)5 and (CdSe)6 core fits well into the (CdSe)28 cage to form an extremely stable network. The theoretical prediction fits well with the experiment results including time-of-flight mass spectra, extended X-ray absorption fine structure (EXAFS), analysis and AFM estimate. This calculation also predicts the similar “magic number” behavior in other II–VI compounds while is less stable for III–V nanoparticles. (One interesting phenomenon they observed is also the appearance of regular QD by simply heating MSQD to a higher temperature, the absorption peak of MSQD remains without shift although the intensity decreases. This indicates the regular QD and MSQD are thermodynamically convertible without the aid of other chemicals. The presence of an intermediate state is still unknown.)

### Solvothermal Synthesis (organic medium) of Semiconductor Nanocrystal Quantum Dots

Solvothermal method (in aqueous medium called hydrothermal) has been extensively utilized for nanoparticle synthesis. However, the earlier efforts to synthesize semiconductor quantum dots are largely restricted by the inferior product properties. Also, large amount of works generated nanoparticles without quantum confinement, without tight control of particle size and morphology. Ref. [1] has provided an introduction until the year of 2004.

Recently, there are a number of efforts reporting the solvothermal synthesis of quantum dots in organic solvents. CdS [22], CdSe and CdSe/CdS core/shell [23], PbSe [24], InP [25], and CuInS_2_ [26, 27] quantum dots have been reported via the approach. Solvothermal could provide evaluated temperature and pressure thus generates unique synthesis condition for nanocrystal growth. The solvothermal method employs similar synthesis method as the normal batch synthesis, usually starting from the mixing of two individual precursors at lower temperature, then increases to the desired growth temperature in a sealed autoclave for the nanocrystal production. In a typical solvothermal growth of PbSe quantum dots [24], Pb precursor was prepared by dissolving Pb(CH_3_COO)_2_ · 3H_2_O in octadecylamine to form a clear solution at 80 °C. Se powder was rapidly added into Pb precursor for a rigorous stir for ~10 min. The mixture was then sealed in a Teflon-lined stainless steel autoclave and maintained at 200 °C for 1.5 h. Different sizes of PbSe nanocrystals could be obtained by varying the initial Pb-to-Se ratio.

Although the synthesized quantum dots show narrow size distribution and quantum confined absorption spectra, the reported photoluminescence efficiency is still unknown or lower than those reported from organometallic or its modified method. A PL quantum yield (QY) of solvothermal synthesized CdSe is around 5–10 % and could reach 18–40 % after a CdS shell was added [23]. This indicates a lower quality of quantum dots possibly originated from higher defects level.

### Aqueous and Hydrothermal Synthesis of Quantum Dots

Quantum dots made from aqueous synthesis is especially attractive for biological application due to their compatibility with water. Also compared to the organic-based synthesis, aqueous synthesis is cheaper, less toxic and more environmental friendly. Unfortunately, the quality of the as-prepared quantum dots has relatively low quantum yield and large size distribution compared to organic approach samples. The additional post-synthesis treatments such as size-selective precipitation, selective photochemical etching, and surface modifications could further improve the quantum dot quality. However, those treatments add additional complexity and cost thus are not desirable for large-scale production.

Representative publications about aqueous synthesis of QD include the material systems of CdSe [28], CdTe [29, 30], CdTe/ZnTe [31], CdTe/CdS [32], CdTe/CdSe [33], ZnS [34], ZnSe and Zn_1 − x_Cd_x_Se alloyed [35], and ZnSe(S) alloyed [36]. Thiols and thioalkyl acids including thioglycolic acid (TGA) and 3-mercaptopropionic acid (MPA) are popular stabilizers used for aqueous synthesized quantum dots. Most of the aqueous synthesis utilized the mixing of two precursors containing the respective anions and cations to react at high temperature. The reaction mechanism follows the similar discussion as the organometallic approach featuring the nucleation and growth stages [37]. A different strategy developed by Li utilized a water–oil heterogeneous system to synthesize nanocrystal and quantum dots [38]. The reaction is based on a general phase transfer and separation mechanism [39]. Synthesis of CdSe [38, 40], ZnSe, Zn_x_Cd_1 − x_Se [38], CdS, PbS, and ZnS [39] quantum dots has been reported from the methodology.

Hydrothermal synthesis uses water instead of organic solvents as the reaction medium. Although numerous papers report the hydrothermal synthesis of nanoparticles, few of them are within the quantum dot category with desired properties needed for applications. The high-quality quantum dots synthesized by hydrothermal method can be found in CdTe [37, 41, 42], CdTe/CdS core/shell [43], CdTe/CdSe core/shell [44], and CdTeS alloyed [45] systems. The hydrothermal synthesis also starts from two precursors containing the cation and anion species, with mixing and heating towards the growth temperature for quantum dot production. In a typical hydrothermal synthesis of CdTe quantum dot, NaBH_4_ was used to react with tellurium in DI water to form the NaHTe as Te precursor. CdCl_2_ and thiol ligand N-acetyl-l-cysteine (NAC; as the surfactant) were dissolved in DI water to form Cd precursor. The pH value of Cd precursor needs to be adjusted to 9.5 by stepwise addition of NaOH at 4 °C. The NaHTe solution was then added into the above Cd precursor at 0 °C with rigorous stirring, and the mixture was subsequently loaded into a Teflon-lined stainless steel autoclave. CdTe quantum dots will form by heating the autoclave at 200 °C for ~1 h. Unlike the reaction in organic medium, pH value is essential for aqueous-based hydrothermal reaction and needs tight control to achieve desirable results. Also, the reaction temperature and precursor ratios are considered important parameters to affect quantum dot formation as well.

Compared to normal aqueous synthesis, hydrothermal method could provide a higher reaction temperature exceeding the boiling point of water and a higher pressure which could favor the formation of nanocrystal. The quality of the synthesized quantum dots has improved significantly in recent years. The PL quantum yield, as an important parameter to evaluate quantum dot quality, is reported to be 68 % for CdTeS alloyed QD [45], 45–64 % for CdTe/CdS, 27.4 % for CdTe [41], and 44.2 % for CdTe/CdSe QD [44] for hydrothermal synthesis approach. The FWHM, indicating the size distribution of the QD, is reported to be ~50 nm for CdTeS alloyed QD [45], ~40–50 nm for CdTe/CdS [43], 40–80 nm for CdTe [41], and ~80 nm for CdTe/CdSe [44]. Although the best value of the QD yield is comparable to the parameters via organic approach, the size distribution is still wider with further optimization needed.

Hydrothermal and aqueous synthesis of nanocrystal quantum dots is relatively new compared to the well-developed organometallic synthesis and its derived method. They have the intrinsic advantages in terms of lower cost, lower toxicity, and “greener” processing. Although attractive material properties such as narrow size distribution and high photoluminescence comparable to organic synthesized quantum dots have been reported, relatively rare device applications have been explored. The real application in energy field requires a more complementary investigation with close interaction with device scientists needed. The formation of functional thin film and the corresponding device performance need further evaluations before concluding them as feasible approaches for energy applications.

### Possible Approaches Towards Large-Scale Synthesis of Semiconductor Nanocrystal Quantum Dots (microwave and flow-through)

Due to the increased demand of quantum dots for various applications, scientists have begun to explore the large-scale synthesis of nanocrystal quantum dots by various means. Although there are a number of companies capable of generating a large amount of quantum dots, they are not discussed here due to the limit of their experiment details. All the discussions below are based on published journal papers and patents available.

Compared to general chemistry, chemical engineering has accumulated extensive theoretical and experiment experience in large-scale synthesis. It is wise to apply chemical engineering guideline to guide our behavior in large-scale synthesis. Most of the syntheses mentioned above are categorized as heterogeneous reactions. In these kinds of reactions, the temperature, pressure, and compositions are obviously important variables. Besides that, the momentum transport, energy (heat) transport, and mass transport are also key variables for large-scale industrial synthesis. The understanding and control of the above parameters are essential to obtain high-quality nanocrystal quantum dots as well as achieving higher yield.

Selection of reactors is important to control the quality and quantity of final products. At initial research stage, batch reaction is obviously the best choice in terms of easy processing and easy control. After the full understanding of reaction mechanism and proof of high-quality nanocrystal production ability, a continuous process will be more preferable for higher production rate and real industrial production. Large-scale production of semiconductor nanocrystal by a continuous flow reactor has been demonstrated by scientists from Japan [46]. The reaction recipe followed the previous batch synthesis using trioctylphosphine oxide (TOPO) as the capping organic ligand and the high temperature reaction solvent [47–50]. The continuous flow reactor is composed of tanks of two starting liquids (ligand TOPO and Cd/Se stock solution, respectively), two feeding pumps, two feeding pipes, one reactor, one heating zone, one condenser, and a collecting glass bottle. The reactor is made of stainless steel and is equipped with a static mixer (screw) for enhancing the mixing of the starting liquids. Starting from the feed pump, the starting liquids (TOPO and the Cd/Se stock solution) went through the feed pipes for preheating before they were continuously fed into the reactor from the feed pumps and pipes. The heating zone is equipped with stainless steel pipes for desired reaction time and temperature. The product was cooled to 70–80 °C (above the melting point of TOPO) by the condenser and finally collected in the collecting glass bottle. In the whole reaction process, dry argon was used to maintain the inside atmosphere. To obtain a high-quality CdSe nanocrystal, the authors have investigated a number of variables. It is determined to be crucial to control a desired reaction temperature at the mixing point of the starting liquids in the reactor. A relatively high reaction temperature of 350 °C is necessary for obtaining high-quality nanocrystal with high photoluminescence efficiency. Also, the quality of commercial TOPO affects the product and adding a small ratio of TDPA helps to control the nanocrystal formation. After the optimization, up to 13 g/h production rate was observed to last for at least 1 h. The bandwidth and the luminescence intensity of the nanocrystal were comparable to batch reaction using the similar approach [47, 48]. There are also a number of works reporting the synthesis of quantum dots via the microfluid reactor [51–53]. Although much smaller in size, the “flow-through” concept could still serve as a useful hint for the future design of flow reactors.

Besides the continuous flow reactor, microwave synthesis is also a plausible approach towards large-scale synthesis. Microwave synthesis features simple operation and potential of large-scale synthesis [54]. The microwave synthesis has enabled production of high-quality colloidal semiconductor nanocrystal both in organic and aqueous media.

For an aqueous medium reaction, a number of monodispersed nanocrystals including CdTe [55], CdTe/CdS/ZnS [56], CdSe [57], ZnSe(S) alloyed [58], and CdSe(S) alloyed [59] have been successfully synthesized by microwave or microwave-assisted synthesis. In a typical reaction of preparing CdTe nanocrystal [56], the CdTe precursor solution was prepared by adding freshly prepared NaHTe solution to a N_2_-saturated CdCl_2_ solution (pH = 8.4) in the presence of the stabilizer 3-mercaptopropionic (MPA). The precursor solution was loaded into a vitreous vessel and heated at 100 °C for 1 min under microwave irradiation before a high-quality nanocrystal was obtained. Some of the best quantum dots synthesized by this approach possess a high photoluminescence quantum yield as well as excellent photostability and favorable biocompatibility [56].

Other efforts have been put on colloidal synthesis of nanocrystal in organic medium. The synthesis method and recipe are similar to the previous reports with the microwave heating replacing the conventional heating. High-quality monodispersed InGaP, InP and CdSe [60], CdTe [61], CdS [62], and CdSe/ZnS [63] nanocrystals have been reported by this method. Microwave dielectric heating enhances not only the rate of formation but also the material quality and the size distribution. The final quality of the microwave-generated materials depends on the reactant choice, the applied power, the reaction time, and the temperature.

In this approach, Strouse and his collaborators emphasized the “specific microwave effect” which will selectively heat certain compounds in the system due to the different polarization of different components. To benefit from the effect, the reactive precursors have to be strong microwave absorbers and the solvent should have no or weak absorption to the microwave. In that case, the polarizable reacting precursor will selectively absorb most of the microwave energy to overcome activation barriers and start the nucleation. Inspection of the cadmium source proved the trend: only the polarizable starting precursors, cadmium stearate (CdSA), CdO, and CdNO_3_ produced nanocrystals while ionic CdCl_2_ had no nanocrystal produced.

The use of microwave irradiation has several unique advantages compared to convective heating. It can selectively heat the target precursor, has good reproducibility, and most importantly is compatible to non-injection approach and could be coupled with a continuous process for large-scale production. In Strouse’s work, they have introduced a near continuous reaction called “stopped flow” approach for nanocrystal synthesis [61]. In such kind of synthesis for CdSe nanocrystal, the two precursors TOPSe and CdSA were delivered into the reaction chamber from separate sources and heated to 190 °C for 4 h to complete the reaction. After the reaction, the mixture-containing product is pumped out to a sealed vessel while the reaction can be continued by pumping new reaching precursors into the reaction vessel. This stop-flow design has demonstrated the yield of nanocrystal for ~650 mg/h.

### Application of Semiconductor Nanocrystal in Energy Utilization

From the introduction of quantum dots [64], they are closely related to semiconductor devices due to their special quantum confinement properties. The current worldwide energy shortage requires renewable energies and energy-efficient techniques. Quantum dots are perfect candidates for answering the challenges in terms of their potential application in solar cell and solid-state lighting/display. Compared to the other applications of quantum dots such as medical labeling, the energy-related applications require much more amount of quantum dots. Thus, the scale-up synthesis of quantum dots aims mainly to the energy utilization, and the production has to be shaped in order to fulfill the requirement for energy applications. The quantum dot application in energy field currently falls in the two main categories: solar cell and LEDs. There are plenty of review papers reviewing the area. For example, Talapin and his collaborators recently provided a comprehensive review of colloidal nanocrystals for electronic and optoelectronic applications [65] during this manuscript preparation. For quantum dot-based solar cell, readers may refer to Refs. [66–68]. For quantum dot-based solid-state lighting, Ref. [69] provides a detailed introduction. As a result, this part is not intended to provide a detailed introduction of quantum dot-based optoelectronic devices. On the other hand, the authors try to integrate the material property with the target application thus provides guideline for the optimization of quantum dots and the future large-scale synthesis in industry.

#### Solar Cells

Solar cells are devices to convert sunlight to electricity by photovoltaic effects. Multiple generations of solar cells have been developed since the 1940s. Solar cells made from crystalline silicon and GaAs show outstanding performances but are limited by the high cost of material and fabrication. Thin film solar cells made from CdTe and CIGS are promising but still suffer from cost issues. Other interesting systems include dye-sensitized and organic solar cells which benefit from their lower fabrication cost and larger flexibility.

The original driving force to use quantum dot comes from the expectation for quantum dot to generate multiple excitons upon absorbing a single photon. This would possibly boost the energy conversion efficiency beyond the traditional Shockley and Queisser limit for silicon solar cells [70]. Besides that, quantum dot combines the benefits of inorganic and organic materials. Compared to conventional inorganic semiconductors, it has low cost, is solution-based, has low temperature processing, is compatible to flexible and large area substrates, and has a larger absorption cross section so that it needs much thinner layer to achieve complete absorption. Compared to organic semiconductors, it features enhanced charge separation efficiency, balanced carrier transport properties, higher PL quantum efficiency (so that more excitons can be generated), and better chemical stability. Besides that, some quantum dot systems (e.g., PbSe) could cover infrared spectrum which is barely utilized by the current available solar cells.

In the solar cell, the generation of electricity involves the processes of light absorption, exciton generation and diffusion, charge separation, transportation, and collection. In most of the quantum dot-based solar cells, quantum dots not only serve as a light-harvesting material but also play multiple roles to help in charge separation and transportation. Three types of solar cells have been reported in this category: quantum dot/metal Schottky junction solar cell, polymer/quantum dot hybrid solar cell, and quantum dot/quantum dot D/A solar cell.

The simplest structure used for quantum dot-based solar cell is the Schottky junction structure. The Schottky barrier is made by contacting the quantum dot layer with a metal electrode. The other side of the quantum dot layer is contacted with ITO layer for the hole transport. Such kind of device features easy structure and processing. Several devices made from the structure have been reported based on PbS [71, 72], PbSe [73], and PbS_x_Se_1 − x_ [74] nanocrystal systems. Due to the small bandgap of the two nanocrystals, those devices are able to utilize infrared part of the solar energy pretty effectively. The nanocrystal thickness ranges from 65 nm to a few hundred nanometers. A ligand exchange process is reported to improve the nanocrystal conductivity and device performance. The nanocrystal layer is made either by spin coating [71] or layer-by-layer dip coating [72, 73] techniques. The power conversion efficiency has been reported to be 2.1 % for PbSe [73], 2 % for PbS [72], and 3.3 % for PbS_x_Se_1 − x_-based devices [74]. More importantly, those devices can effectively collect infrared light with a monochromatic power conversion efficiency (MPCE) of 4.2 % reported in the infrared [74]. Notice that the PbS quantum dot used in the Schottky junction structure is determined to be p-type owing to the high ratio of hole-to-electron mobility [75]. Most recent development on infrared absorbing quantum dots and solar cells is updated in review articles [92, 93].

Since semiconductor nanocrystals are good hole transport materials but not good at electron transport, a quantum only layer will likely have an unbalanced electron/hole transport and restrict the final device performance. To solve the issue, a quantum dot/conjugated polymer mixture layer is proposed to serve as the active layer. P3HT and MEHPPV are the two most popular polymer materials to be selected due to their superior electron transport and light-harvesting capabilities. The blend facilitates the charge separation and the generation of photocurrent.

Two structures have been widely used in quantum dot/conjugated polymer blend solar cell: bilayer configuration of n-type quantum dots and p-type conjugated polymer and a so-called “bulk heterojunction” structure.

Since the generated excitons (upon light absorption) have a diffusion length of ~10 nm, the bilayer configuration will have to limit the thickness of each active layer less than 20 nm for an effective charge carrier collection. This, on the contrary, will restrict the light absorption. A solution is to build an interconnected network of quantum dot and conjugated polymer called “bulk heterojunction” structure. In such kind of structure, donor and acceptor domains have the range close to the exciton diffusion distance so that most excitons can reach the donor and acceptor interface in a thicker device. Meanwhile, percolation pathways are required in both donor and acceptor domains for charge carriers to be transported in each individual domain. An interconnected network meeting the two requirements could achieve better light collection, charge separation, and transportation in a much thicker device than bilayer structure device. Devices with bulk heterojunction structures have better device performance reported. The best material system utilizes CdSe nanorod and P3HT and has achieved higher power conversion efficiency.

Due to stability concern of polymer/organic materials, efforts have been put on developing pure inorganic quantum dot solar cell. One representative work has been done by Alivisatos and his colleagues. They use a CdSe/CdTe nanorod system and a bilayer heterojunction structure [76]. The CdSe and CdTe nanorods by their energy alignment form a donor/acceptor pair. The films of CdTe and then CdSe were sequentially spin casted on ITO glass coated with 2 Å alumina. CdTe and CdSe will serve to transport holes and electrons, respectively. The charge separation happens not only at the donor/acceptor interface but also along the length of the nanorod since little quantum confinement is applied on the direction. Free charge carriers can be generated all along the thin film. The power conversion efficiency is reported to be 2.9 %. Without the organic materials involved, the device shows a better stability in the air.

Quantum dots have also been widely used in solar cells for sensitization. Semiconductors with visible light absorption can serve as sensitizers to transfer electrons to large bandgap semiconductors such as TiO_2_ and SnO_2_. People have built up such kind of solar cells similar to dye-sensitized solar cells. A detailed review is available by Kamat [77]. A power conversion efficiency of 1 % has been reported for CdSe-sensitized TiO_2_ solar cell with a cobalt (II/III)-based redox system [78].

#### More Discussion of Different QD Solar Cell Developments

There are also other solar cell devices utilizing semiconductor nanoparticles. They are not discussed here either because the particle size exceeds the Bohr radius so no quantum confinement is affected or because the nanoparticles are sintered and used as bulk materials.

Theoretically, a suitable solar cell material system has to satisfy the following requirements: a bandgap matching the solar spectrum, high electron and hole mobility, long lifetime of charge carriers, good chemical stability, relative abundance of the composites, and relatively low melting points.

For the current applications, a few concerns need to be considered for quantum dots to be used in solar cells. First of all, the quantum dot should have strong absorption starting from infrared or visible. A quantum dot system with too large bandgap will not be able to utilize most parts of the solar spectrum while systems with too small bandgap will have trouble with limited open-circuit voltage. Second, the quantum dot should have high electron mobility and long charge carrier lifetime to ensure efficient charge transportation. Although the intrinsic property of the colloidal quantum dots determines the transport property, surface ligands need to have more concern in the practical experiment design. Surface ligands are required to protect quantum dots, prevent agglomeration, and passivate surface from the solution to thin film form. In the device, appropriate ligands help to reduce the surface defects which will trap the charge carriers and reduce the charge transportation process. On the other hand, surface ligands are less conductive or insulating materials which have negative effects on the device conductivity. To reduce that, appropriate ligands need to be selected during synthesis. The best ligands should be able to be removed upon heating or UV treatment after forming the thin film. Otherwise, surface ligands should be short and conductive or could be exchanged to the desired type. The long and insulating ligands should be avoided if post-treatment is not available for conductivity enhancement.

Compound toxicity and abundance could also be a factor when it comes down to mass production of solar cell materials. Toxicity of cadmium and lead has been a concern since the initial development of quantum dots. The issue could be managed if appropriate package and post-treatment are applied to the products. Most of the compounds used in the current quantum dots are abundant for R&D and practical applications. Tellurium might be a concern as it is listed as the nine rarest “metals” in the earth. So the mass production of tellurium-based quantum dots will be considered with caution, which would need to be concerned by CdTe thin film solar cell as well.

Different morphologies of semiconductor nanocrystals also affect the solar cell device performance. In quantum dot-based solar cells, charge separation happens at the donor/acceptor interface, and charge carriers are transported in quantum dot phase by hopping and tunneling. If semiconductor nanocrystals have one or two dimensions of freedom (like nanorod), excitons can be separated along the free dimension and free carriers can also be transported along the direction. A more ideal structure will be an organized array of nanocrystals perpendicular to the electrode surface. This will provide more interface area for charge separation and significantly improve the charge transport efficiency. Post-synthesis techniques need to be designed to achieve such kind of structure.

#### Solid-State Lightings and Displays

Quantum dot application in solid-state lighting and color display is very close to commercialization due to the superior optical properties of the quantum dots. Compared to its immediate precedence organic semiconductor-based optical devices, it is chemically more stable and enables purer color emission. Compared to the traditional semiconductor device, it is significantly lower in fabrication cost and compatible to flexible large substrates.

The most attracting optical property of quantum dot in lighting is its narrow band emission. The FWHM of the emission peak (also the first excitation peak of absorption spectrum) is related to the size distribution of the obtained quantum dot. Regular quantum dots with appropriate size selection process could have a size distribution corresponding to a FWHM of ~20 nm while the MSQD has a FWHM as narrow as 10 nm.

Currently, quantum dot application in solid-state lighting mainly lies in the phosphor usage in white LED applications. The current main stream technology for white light emission couples blue LED and color-converted phosphors to produce white light. Commercially available phosphors show high quantum efficiency of above 90 % for yellow (yttrium aluminum garnet, YAG) and 80 % for red (CaAlSiN, CASN). Quantum dots need more improvement in solid-state form to match those values. However, quantum dots could still serve as useful substitutes or add-ons of those phosphors for enhanced performance due to several reasons. First of all, their size-tunable emission property makes the multiple color emission easy to achieve without employing multiple compositions. This could reduce the chemical compatibility and aging problem when intermixture of different compositions is inevitable. Second, the continuous tunable color of quantum dot enables them to be added in to reach a high color rending index (CRI) value. By adding more phosphors with different colors, the white light source shows richer color, broader emission spectrum, and higher CRI value. Third, nanocrystals are very light and tiny so they could relief the phosphor settling problem due to the gravity. Finally, commercially available phosphors could be restricted by the abundance of rare earth elements. Quantum dot phosphor, if selected with right material systems, will not face such kind of problems.

#### White Light Emission by Depositing Red-Emitting CdSe/ZnS QDs on a Blue/Green InGaN/GaN Quantum Well LEDs

CdSe MSQD has been reported as a potential phosphor [79]. The used MSQD has a significant amount of tail emission all over the visible range and is used to simulate the white emission. By excitation from a UV light, a chromaticity coordinate of (0.324, 0.322) and a high CRI of 93 have been reported from the emission. A potential problem of this approach originates from its emission mechanism. Most part of the spectrum comes from the defect emission which will be hard to repeat for industry processing, and the brightness will be greatly restricted as well.

The requirements of quantum dot to serve as phosphor is mainly the emission wavelength, quantum efficiency, and the chemical stability. As an initial step, phosphors with different colors (compared to the available phosphor) can serve as add-ons to boost white light emission reaching higher CRI value. As a second step to replace main stream phosphors, quantum efficiencies of quantum dot phosphors need to reach comparable values with current competitors. The chemical stability at elevated temperature is also a key parameter to be evaluated which could be improved by selecting appropriate surface ligands. Overall, this is the application of quantum dots closest to commercialization due to the simplicity of achieving photoluminescence.

The abovementioned quantum dot phosphors utilize an absorption-reemission process to emit light. This process is not efficient enough than another process called Förster resonance energy transfer (FRET). An effective FRET requires two conditions: a short center-to-center separation distance between the two interacting composites and a spectral overlap between donor emission and acceptor absorption. The second condition is usually fulfilled for the quantum dot phosphor coupled with traditional semiconductor LEDs. But to satisfy the first condition (distance within 10 nm), special architectures are needed to bring quantum dot close enough to the emission media. Klimov et al. put a monolayer of CdSe/ZnS quantum dots on top of the InGaN quantum well to achieve a ~55 % light conversion efficiency [80].

Most of the quantum dots used in LED applications utilize electroluminescence rather than photoluminescence. The LED device based on quantum dot electroluminescence is called QD-LED. Extensive research efforts have been put on the device during the last decade. QD-LED utilizes a similar structure as OLED (organic LED) and is composed of two electrodes for charge injection, two charge transport layers (for electron and hole transportation), and the quantum dot emission layer. The electrodes are ITO for hole injection and metal for electron injection for most of the cases. The charge transport layers are usually conducting polymer or organic molecules which help to transport electron and holes into the emission layer.

Electroluminescence of quantum dots usually comes from two mechanisms: direct charge injection and Förster resonance energy transfer. In the direct charge injection case, electron and hole will be injected from two sides of the electrodes and then trapped in quantum dots due to the energy alignment between electron transport layer (ETL), emission layer, and hole transport layer (HTL). The trapped electron and hole will form exciton accordingly and then recombine radiatively to emit light. In the Förster energy transfer case, excitons will form on organic molecules close to QD, interstitial spaces, and voids in the QD layer. Due to fulfillment of the two requirements (spatial distance close enough and energy overlap), those excitons will likely move to lower energy QD sites via FRET then undergo the radiative recombination.

Successful QD-LEDs were first demonstrated by Alivisatos group in 1994 [81]. TOPO-TOP-capped CdSe QDs were used as the emission layer, and poly(p-phenylenevinylene) (PPV) was used as hole transport layer. Different color emissions from red to yellow were demonstrated although the external quantum efficiency is pretty low.

The low quantum efficiency of early works is partially due to the imbalanced carrier injection into the emission layer. The problem was addressed adding an electron transport layer tri(8-hydroxyquinoline) (Alq3) to form the sandwich structure [82]. With the new device structure, Bawendi and Bulovic group have reported an external quantum efficiency of >2 % and brightness of over 7000 cd/m^2^ on their optimized device [83]. Besides the sandwich structure, those devices also feature monolayer thickness of QD emission layer and a phase separation technique to separate emission layer and HTL during spin casting process.

The highest device brightness by far was reported by Sun et al. in 2007 [84]. They used the similar device structure but optimized QD emission layer thickness based on different QD size, structure, and emission colors. The maximum brightness was reported to be 9064, 3200, 4470, and 3700 cd/m^2^ for red, orange, yellow, and green QD-LEDs. A lifetime (a slow decay to 50 % of initial value after over 300 h) was reported at a brightness of over 1100 cd/m^2^. Those QD-LEDs also show low turn-on voltage, improved electroluminescence efficiency, and defect-free emission on relatively large surface areas.

Multi-color QD-LEDs can find their applications in color displays. But among those blue emission is the most difficult to achieve due to the difficulties in obtaining high-quality blue QD and the low sensitivity of human eyes to blue light. QD size shrinks according to the blue shift of emission wavelength. As a result, QD with blue emission has the smallest size and usually forms at the initial stage of QD growth. Thus, more defects are expected for blue QD which results in lower quality. Also, the luminous efficacies (specifying the average sensitivity of human eyes to light with different wavelength) of blue light are significantly lower (0.02–0.2) than green and red (0.8–1), requiring much higher radiant power for blue light to achieve a brightness comparable to its red and yellow competitors.

Up to now, the best blue QD-LED was reported by Tan et al. [85]. They used CdS/ZnS core/shell QD to build the device, and ETL is omitted to avoid the extra organic emission from Alq3. The device demonstrates pure blue emission with high brightness (1600 cd/m^2^), a low operating voltage (~5.5 V), and a narrow bandwidth (~20 nm). Recent progress on the QD-LEDs is updated in review articles [94, 95].

Besides the single color QD-LED, QD electroluminescence can also be used in white LED. Emission layer is usually composed of several kinds of QD or a mixture of QD with organic semiconductors. For example, a polymer poly[(9,9-dihexyloxyfluoren-2,7-diyl)-alt-co-(2-methoxy-5-phenylen-1,4-diyl)] (PFH-MEH) for blue, a CdSe/ZnS QD for red color, and Alq3 for green have been combined together to achieve white emission [86]. In another work, three different types of QDs (CdSe/ZnS for red, ZnSe/CdSe/ZnS for green, and CdZnS alloy for blue) were used to form a mixed monolayer of QD for the white light emission. Besides N,N′-bis(3-methylphenyl)-N,N′-bis(phenyl)benzidine (TPD) as the hole transport layer and Alq3 as electron transport layer, an additional 3,4,5-triphenyl-1,2,4-triazole (TAZ) was added between QD emission layer and Alq3 as hole block layer. Blue QD has the lowest quantum yield (40 %) among the three colors. Furthermore, blue emission is unfavorable compared to red and green by both direct injection (higher energy barrier) and energy transfer (less spectral overlap with TPD PL). To compensate and achieve balanced emission, a much higher blue QD ratio was used in the mixed layer. The device shows a color rending index of 86 which is comparable to commercial white LEDs with a maximum brightness of 830 cd/m^2^.

The white QD-LED is much more complicated than single color QD-LED due to the involvement of much more material systems and interactions in between. In the three-color mixing QD-LED example shown above, not only direct charges into QDs and energy transfer from organic molecules to QDs need to be considered but also energy transfers between QDs (from blue to green, red and green to red) and from QD to organic molecules (blue QD to Alq3) are important factors. A detailed understanding of the whole system is the prerequisite for obtaining a white light source based on mixed QD electroluminescence.

Furthermore, there are a few intrinsic problems needed to be considered for white QD-LED design. Faster degradation rate of organic semiconductors (OSC) will bring aging problem of the hybrid OSC/QD system and result in undesired spectrum shift over time. For mixing QD emission device, QD with high emission purity is not desired due to the low CRI value it will bring. The complication brought by more material system and additional interactions also adds more technical challenges than single color QD-LEDs. Although the white QD-LED has a long way to commercialization and even cannot compete with white LED using QD as phosphor, it also has its unique potential in terms of offering large area and flexible lighting source which will render it an interesting direction to be explored in the next few decades.

Material degradation and electrode contact are two limiting factors of QD-LED lifetime. To address the problem, efforts have been put on building pure inorganic QD-LED. Such kind of device has been demonstrated by Bulovic and Bawendi group at MIT [87]. In the device, the hole and electron transport layers are replaced by p-type NiO and n-type ZnO:SnO_2_. The device demonstrates a peak brightness of 1950 cd/m^2^ and an external quantum efficiency of 0.1 %. A big challenge of the work is to select an appropriate inorganic n-type semiconductor material to serve as ETL without damaging QD emission layer. Room temperature radiofrequency (RF) sputtering was selected as the technique for depositing ETL and HTL to keep the QD layer intact. The two charge transport layers were selected to have similar free-carrier concentration and energy band offset to the QD for balanced charge injection. The resistivity and surface roughness of the two layers were also tuned during deposition for good charge conductivity and electrical contact. The potential benefits of this approach are the following: it provides large selection of materials and tunability of conductivity and energy band during processing.

As claimed before, QD-LEDs are compatible to large and flexible substrates due to its solution processing property. An initial work of flexible QD-LED was demonstrated on ITO-coated poly(ethylene-terephthalate) (PET) substrates [88]. The device structure is similar with comparable brightness reported as device based on rigid substrates. The device flexibility is demonstrated by remaining its performance until bending radii reduces down to ~5 mm. This value is below the bending limit of many possible applications and sufficient for large, roll-up flat panel displays.

Overall, LEDs based on QD electroluminescence face more technical challenges than devices using photoluminescence. But it provides much more freedom in device design and has more potential to achieve better performance. Since it no longer requires the pumping source made from traditional LED chip, the QD benefits could be fully realized. The future of QD-LEDs can be predicted by comparing it with OLEDs due to many similarities these two types of LEDs share. The first organic electroluminescence device was demonstrated in 1987 [89] and OLEDs have been extensively studied during the last decade. OLED products have been commercialized in cell phone, display, and many other applications. The QD-LEDs have some intrinsic benefits over OLEDs (chemical stability, emission properties, etc.) and can benefit from OLED technique due to their similar structure, so the commercialization of QD-LEDs should be seen in the near future.

There are a few common properties to be shared between QDs used in solar cell and those in LEDs: they should have good conductivity and chemical stability, abundant amount of supply, and controllable toxicity. QDs in LEDs undergo more hush conditions due to the elevated temperature from high injection current.

1. A high quantum efficiency (high PL usually leads to high EL) indicates good recombination and low defect level.
2. There are several reasons for reduced EL efficiency compared to PL efficiency: liquid to solid lost (one magnitude), inefficient and imbalanced charge injection.
3. Emission bandgap can be designed to fulfill specific wavelength.
4. The narrower the bandwidth the better, for single color emission and color display. But for white LEDs, high color purity is not desirable due to the low CRI it will bring.
5. Good conductivity is desirable.
6. Good chemical and thermal stability are required due to high current and elevated operating temperature. Good encapsulation could help maintain the stability. The encapsulation technique developed from OLEDs could be applicable for the LEDs.
7. Low toxicity is necessary for environmental compliance by replacement of CdSe QDs with non-toxic elements.

Finally, surface engineering may play a significant role for better charge injection, lower nonradiative recombination, and improved thermal stability. Design of novel material system with comparable or better performance than cadmium-based QD is needed.

## Conclusions

This paper has reviewed the various experimental methods in the laboratory scale. It appears that the non-injection one-pot synthesis route seems to have the promise for scale-up engineering development for larger scale production with a chemical engineering stir-tank reactor. This paper also reviewed a few limited literature developments on scale-up engineering development and green approaches. Applications in energy such as solar cells and solid-state lightings demand quantum dots as “commodity” materials. The challenges on large quantity production while maintaining the high optical quality of quantum dots need to be addressed.

## Abbreviations

CRI: color rending index
ETL: electron transport layer
FRET: Förster resonance energy transfer
HTL: hole transport layer
PL: photoluminescence
QDs: quantum dots
LEDs: light-emitting diodes
OLEDs: organic light-emitting diodes
